# Advanced Electroanatomic Mapping with the TriDeca™ Catheter in Left Ventricular Assist Device–associated Ventricular Tachycardia: A Report of Two Cases

**DOI:** 10.19102/icrm.2026.17042

**Published:** 2026-04-15

**Authors:** Chandler O’ Leary, Alexander Bosley, Ali Saad Al-Shammari, Shahman Shahab, Robert Spalding, Haider Al Taii

**Affiliations:** 1 Department of Internal Medicine, University of Texas Medical Branch, Galveston, TX, USA; 2 Department of Internal Medicine, Baghdad Teaching Hospital, Baghdad, Iraq; 3 Cardiac Ablation Solutions, Abbott Laboratories, Houston, TX, USA; 4 Department of Cardiology, Aultman Hospital, Canton, OH, USA

**Keywords:** Catheter ablation, high-density mapping, LVAD, TriDeca catheter, ventricular tachycardia

## Abstract

Ventricular tachycardia (VT) in patients with left ventricular assist devices (LVADs) poses significant procedural challenges for catheter ablation due to altered cardiac anatomy, mechanical interference, and hemodynamic instability. High-density (HD) mapping technologies may improve procedural efficacy and safety in this complex population. We present two cases of drug-refractory monomorphic VT in patients supported by HeartMate 3™ LVADs (Abbott, Chicago, IL, USA), successfully managed using the TriDeca™ HD linear mapping catheter (Stereotaxis, St. Louis, MO, USA). In both cases, precise localization of slow conduction zones and targeted substrate ablation resulted in sustained arrhythmia suppression without recurrence during follow-up. These cases highlight the value of advanced mapping systems in overcoming LVAD-related limitations and optimizing ablation outcomes.

## Introduction

Ventricular tachycardia (VT) in patients supported by left ventricular assist devices (LVADs) represents a high-risk arrhythmogenic scenario associated with significant morbidity, recurrent hospitalizations, and reduced quality of life.^1^ The pathogenesis of VT in this population is multifactorial and includes pre-existing myocardial scar, surgical trauma during LVAD implantation, mechanical irritation from the inflow cannula, and altered ventricular geometry. These structural and electrophysiological abnormalities contribute to the formation of re-entry circuits and focal triggers, often leading to recurrent VT episodes and frequent implantable cardioverter-defibrillator (ICD) discharges despite optimal anti-arrhythmic therapy.^2^ Catheter ablation has emerged as a vital therapeutic option in LVAD patients with drug-refractory VT. However, the procedure is technically challenging due to distorted myocardial anatomy, particularly near the apical and septal regions, and limited catheter maneuverability caused by the presence of the LVAD cannula. Hemodynamic instability during sustained arrhythmia often precludes activation or entrainment mapping, necessitating a substrate-based approach. Additionally, electromagnetic interference and mechanical artifacts from the LVAD system can compromise electrogram fidelity, further complicating ablation precision.^3^ Advanced electroanatomic mapping technologies are essential to overcome these limitations. High-density (HD) mapping catheters, such as the TriDeca™ catheter (Stereotaxis, St. Louis, MO, USA), offer enhanced signal resolution, greater electrode coverage, and improved flexibility to navigate the complex post-surgical anatomy. These features enable precise delineation of scar borders, identification of slow conduction zones, and effective targeting of critical isthmuses during substrate ablation.^4^ We present the cases of two male patients with nonischemic cardiomyopathy supported by a HeartMate 3™ (HM3) LVAD (Abbott, Chicago, IL, USA) who developed recurrent refractory monomorphic VT and were successfully treated with VT ablation using the TriDeca™ catheter, thus illustrating its utility in overcoming procedural challenges and achieving arrhythmia control in this complex population.

## Clinical presentation

### Case 1

**Figure 1:**
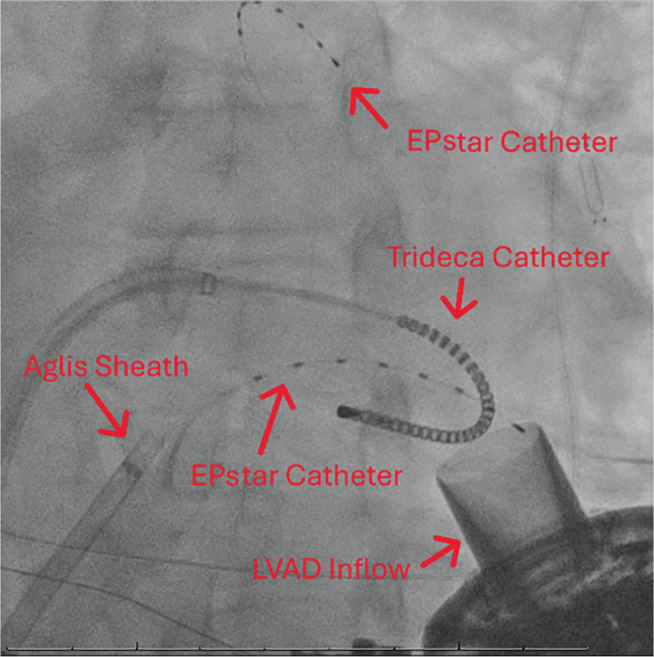
Fluoroscopic view of catheters for case 1. Note that the TriDeca™ catheter is curled in a fashion to prevent suctioning into the left ventricular assist device inflow. *Abbreviations:* LVAD, left ventricular assist device.

**Figure 2:**
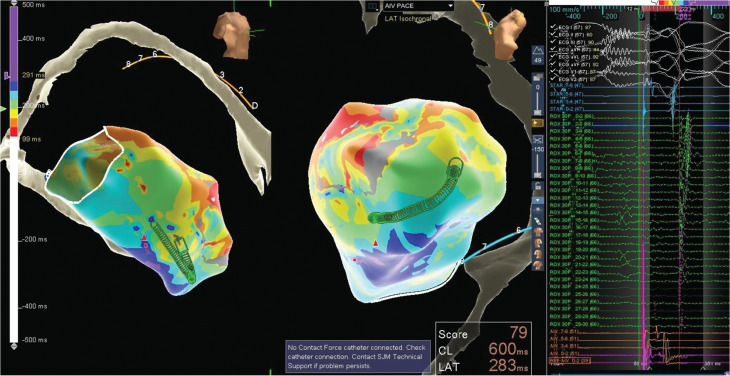
Left ventricular mapping for case 1.

A 63-year-old man with a history of nonischemic heart failure with reduced ejection fraction (HFrEF) status-post (s/p) ICD and HM3 implantation, pulmonary embolism, and paroxysmal atrial fibrillation, who had a recent ICD firing due to VT and was discharged on amiodarone, presented with recurrent ICD firing. An electrocardiogram revealed the VT to be monomorphic. Given the recurrent VT despite anti-arrhythmic therapy, the patient was taken for VT ablation. Access was obtained via the right common femoral vein and right common femoral artery. The coronary sinus was cannulated using a large-curved Agilis™ sheath (Abbott), and two EPstar catheters (Boston Scientific, Marlborough, MA, USA) were placed in the coronary sinus for recording of the lateral left ventricle (LV) and the anterior interventricular branch. Fluoroscopy and intracardiac echocardiography (ICE) were used to guide transseptal puncture. After puncture, the ICE catheter was placed in the right ventricle (RV) during the rest of the procedure to monitor for pericardial effusion. An HD mapping catheter (TriDeca™ HD linear catheter) was advanced into the LV for electroanatomic mapping using the EnSite™ X EP System **(Figures 1 and 2)**. Electroanatomic mapping of the LV was performed during LV pacing at a cycle length of 600 ms using the TriDeca™ catheter. Electrograms were annotated to the onset of the local near-field ventricular signal relative to the paced QRS complex (0-ms reference). Slow conduction was localized to the posterior LV septum, where bipolar voltage mapping defined dense scar as <0.5 mV and the border zone as 0.5–1.5 mV. This mapping revealed a narrow corridor of abnormal low-voltage electrograms within the posterior septal scar, consistent with a slow conduction channel targeted for ablation. An irrigated ablation catheter was advanced and positioned along this region, and radiofrequency (RF) energy at 35 W resulted in the elimination of the slow conduction zones. After ablation, repeat programmed stimulation using the pacing protocol failed to re-induce VT. After removal of all lines, hemostasis was achieved. Post-procedure, the patient had some bradycardia and respiratory failure requiring bi-level positive airway pressure; thus, he was observed in the cardiac intensive care unit. The patient was bridged back to warfarin and discharged without further issue. Device interrogation at 4 months showed no new ICD discharges or episodes of VT, only some asymptomatic atrial arrhythmias.

### Case 2

A 54-year-old man with a history of nonischemic HFrEF s/p ICD placement, paroxysmal atrial fibrillation, and monomorphic VT s/p ablation presented for ICD firing. Device interrogation found ventricular fibrillation that was terminated with defibrillation. The patient was also found to be in cardiogenic shock on admission. He was initially started on milrinone, which triggered worsening nonsustained VT and was therefore discontinued. Hemodynamic support was escalated with the placement of an Impella CP® (Abiomed, Danvers, MA, USA). Despite this, the patient progressed to VT storm requiring multiple bouts of anti-arrhythmics and intubation with sedation for treatment. His VT focus was monomorphic and scar-mediated with a likely re-entry mechanism. The patient was taken for epicardial VT ablation followed by HM3 placement immediately after.

**Figure 3:**
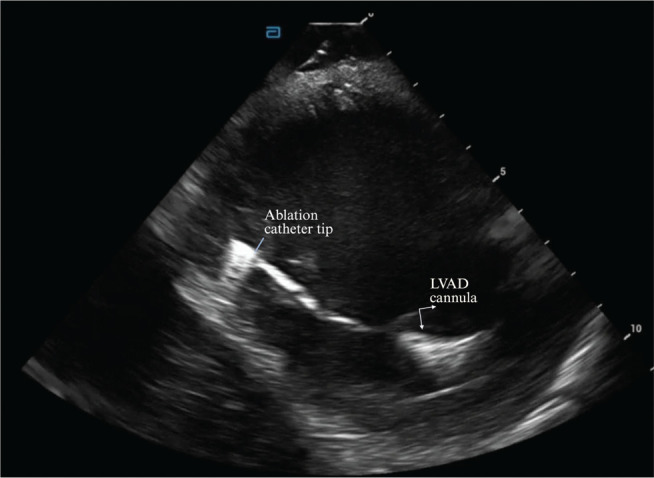
Intracardiac echocardiography image demonstrating the ablation catheter tip positioned adjacent to the left ventricular assist device inflow cannula within the left ventricle. *Abbreviation:* LVAD, left ventricular assist device.

**Figure 4:**
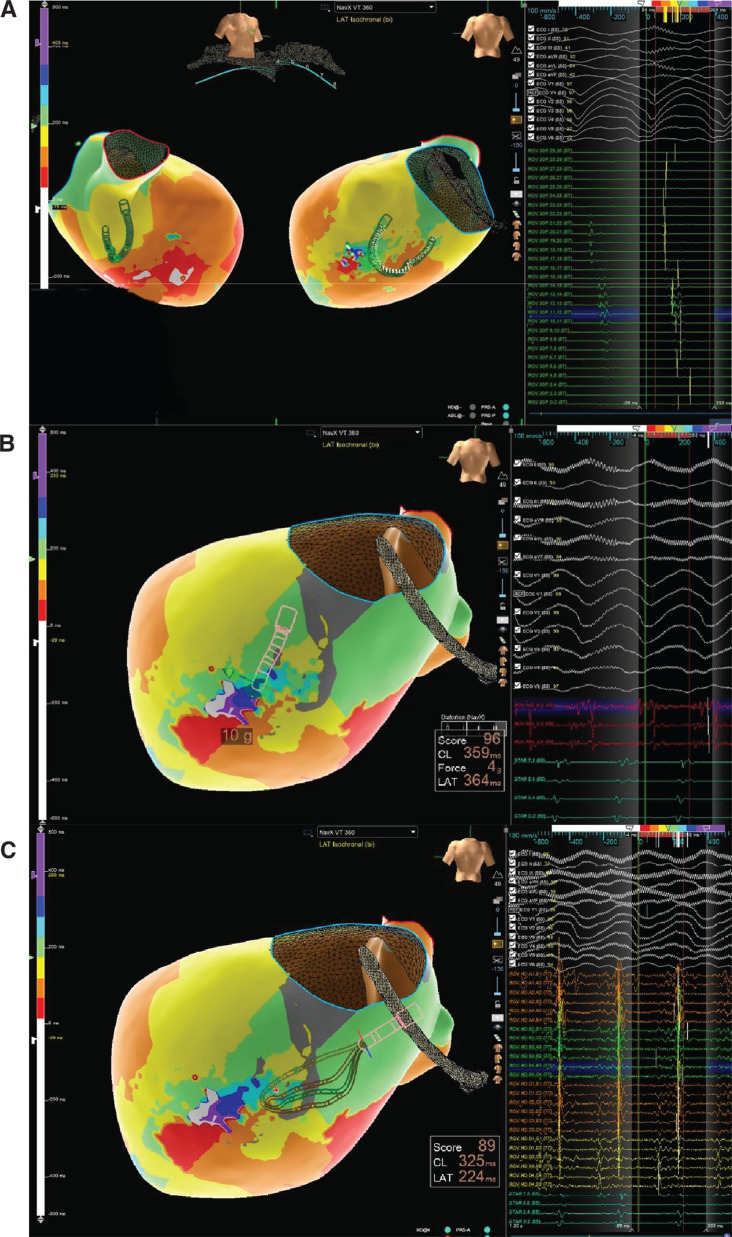
Left ventricular mapping for case 2. **A:** Three-dimensional Left ventricle electroanatomic map in the HeartMate 3™ left ventricular assist device recipient (case 2), demonstrating overall left ventricular geometry and cannula position. High-density mapping with the linear TriDeca™ catheter defines the cannula-adjacent substrate and areas of low voltage/late activation in the basal and mid-ventricle. Surface electrocardiogram leads and intracardiac electrograms from the mapping and ablation catheters are displayed on the right. **B:** Local activation time isochronal map acquired with the TriDeca™ catheter and ablation catheter during induced monomorphic VT (CL, 359 ms). Isochronal crowding within the cannula-adjacent inferoseptal region highlights a slow conduction channel that was targeted for ablation. The ablation catheter is positioned at this late activation site, and representative electrograms from the same region are shown on the right. **C:** Local activation time isochronal map isochronal map recorded during the same VT (CL, 325 ms) demonstrating combined mapping with the Advisor™ HD Grid and TriDeca™ catheters around the left ventricular assist device inflow cannula. The HD Grid and TriDeca™ catheters sample the basal posterolateral left ventricular border zone and cannula-adjacent region simultaneously, illustrating the high-density near-field recordings obtained with the linear TriDeca™ catheter and its complementary role alongside the grid catheter.

The hospital course was thereafter complicated by RV failure requiring temporary placement of a right-sided Impella device and an epoprostenol drip. The patient continued to have episodes of hemodynamically unstable VT, and therefore repeat ablation was performed. Access was obtained via the right common femoral vein. An 8.0-Fr long ICE catheter **(Figure 3)** was introduced under fluoroscopic guidance to facilitate transseptal puncture and was left in place throughout the procedure to monitor for pericardial effusion. Activation mapping (seen in **Figure 4**) was performed during induced monomorphic VT (cycle lengths, 359 and 325 ms), with electrograms annotated to the onset of the local near-field ventricular signal relative to the surface QRS (0-ms reference). Mapping was performed using a TriDeca™ mapping catheter with the EnSite™ X EP System, localizing the area of slow conduction to the basal posterior lateral region of the LV **(Figure 4)**. Ablation was performed using an irrigated TactiFlex™ catheter (Abbott), but the tachycardia recurred with pacing stimulation using the S1–S2 protocol. An HD Grid catheter (Advisor™ HD Grid Mapping Catheter, Sensor Enabled™; Abbott) was then used to repeat mapping, in which the same foci of arrhythmia were identified. RF ablation was repeated with a longer duration, and ICE was used to monitor catheter position relative to the LVAD inflow cannula, assess apparent lesion depth, and screen for microbubble formation or early signs of steam pop or perforation while delivering effective lesions. Afterward, the S1–S2 protocol was unable to induce arrhythmia, but the S1–S2–S3 protocol was able to induce VT again at a faster cycle length. A final ablation successfully terminated the arrhythmia, and no further VT episodes occurred during the remainder of the patient’s recovery.

## Discussion

Catheter ablation of VT in LVAD-supported patients presents unique challenges. We describe two cases of VT in patients with HM3 support. Both cases demonstrate that the use of an HD, linear TriDeca™ catheter offers both high-resolution electroanatomic mapping and procedural safety, even in the complex context imposed by an HM3 LVAD. The combination of precise substrate identification and targeted ablation led to sustained arrhythmia suppression. These cases highlight both the feasibility and limitations of current ablation approaches in HM3-supported patients and demonstrate how tailored mapping strategies can address arrhythmogenic substrates near the LVAD cannula. The presence of a continuous-flow LVAD complicates both catheter maneuverability and electrogram fidelity due to electromagnetic interference, ie, background noise, and altered ventricular loading. This interference can obscure electrogram detail, degrade mapping resolution, and complicate precise substrate identification. This issue is particularly relevant in HM3 devices, where magnetic levitation and centrifugal flow design may contribute to greater electrical noise and artifacts during mapping compared to earlier-generation devices such as HeartMate II™.^5^ However, the TriDeca™ catheter, with closely spaced 1-mm electrodes and high signal fidelity, allowed for precise electroanatomic delineation even in the presence of device-related noise. Its enhanced resolution was critical in accurately identifying slow conduction zones and mapping scar border zones despite the HM3’s electromagnetic artifact.

In our experience, the linear TriDeca™ catheter offered several electrophysiological advantages over conventional duodecapolar or standard ablation catheters in this setting. Its 1-mm, closely spaced electrodes provided high-fidelity near-field recordings with minimal far-field contamination, enabling detailed visualization of late and fractionated potentials within the heterogeneous scar. In case 1, this resolution helped delineate a narrow corridor of delayed activation within the posterior septal scar corresponding to the slow conduction substrate of the clinical VT. In case 2, TriDeca™ mapping complemented HD Grid mapping by allowing the TriDeca™ catheter to be positioned along the basal LV wall and away from the HM3 inflow cannula while still providing continuous multi-bipolar coverage across the cannula-adjacent border zone.

In addition to its superior resolution, the TriDeca™ catheter offers distinct mechanical advantages in the LVAD population. Its linear structure allows it to be gently curled within the ventricle, reducing the risk of catheter entrapment or suction near the inflow cannula. This mechanical feature improves safety during LV mapping, particularly in the apical region, and permits broader contact with the endocardial surface for more rapid and simultaneous multi-point recording. Compared to traditional point-by-point tip-based systems, the TriDeca™ catheter allows efficient multisite mapping over broader myocardial segments without compromising safety or resolution. Furthermore, our approach aligns with recent recommendations emphasizing the utility of substrate-based ablation in LVAD recipients, particularly when hemodynamically stable VT is not inducible or poorly tolerated.^5^ This is supported by Sacher et al., who found that ablation was feasible and effective even shortly after LVAD placement, with the majority of VTs arising from intrinsic myocardial scar rather than cannula-associated circuits.^6^ In case 1, mapping during LV pacing and substrate modification led to the successful elimination of a slow conduction zone in the posterior septum, whereas case 2 required both endocardial and epicardial mapping with repeat ablation to control VT storm. Notably, the short-term success rates of VT ablation in LVAD patients have been reported to range between 77% and 86%, with recurrence rates varying widely from 15%–86%.^5^ In terms of outcomes, the post-ablation clinical trajectory of our patient mirrors findings from larger studies. A significant proportion of LVAD patients treated with catheter ablation experience substantial arrhythmia reduction or complete freedom from recurrence. Bergau et al. reported that, among nine patients undergoing ablation, four remained free of any VT relapse, while others had manageable recurrences, often treated effectively with antitachycardia pacing.^7^ Case 1 demonstrated complete freedom from VT or ICD therapies at 4-month follow-up, while case 2 required a more intensive recovery following initial and repeat ablation but ultimately had resolution of unstable VT in the context of ablation with the HM3 in place. This underscores the clinical value of ablation not only as a therapeutic endpoint but also as a bridge to enhanced quality of life. Finally, retrospective series have shown that a subset of VT circuits may localize near the LVAD cannula, particularly in ischemic cardiomyopathy, with Hohendanner et al. reporting that >30% of VTs in their cohort originated near the cannula,^8^ further supporting the need for precise anatomic delineation and safe catheter control during ablation procedures. In some cases, as illustrated by Izumida et al., VT circuits may involve biventricular or epicardial components traversing the interventricular septum or RV apex,^9^ reinforcing the importance of high-resolution mapping in complex scar-related arrhythmia substrates. In case 2, epicardial access, Impella support, and incremental pacing protocols were necessary to localize and eliminate a difficult-to-induce VT focus, further demonstrating the adaptability of HD mapping techniques across clinical scenarios. In both cases, the use of a linear catheter like the TriDeca™ may facilitate mapping in cannula-adjacent regions without risking suction injury or signal dropout.

## Conclusion

As illustrated by these two cases, catheter ablation remains a feasible and effective strategy for drug-refractory VT in HM3 LVAD recipients when combined with advanced HD mapping. The TriDeca™ linear catheter provided high-resolution definition of scar borders and slow conduction channels while allowing safe navigation around the inflow cannula, enabling targeted substrate modification despite distorted ventricular anatomy and device-related noise. Both patients experienced durable suppression of VT without recurrent ICD therapies, suggesting that HD linear mapping catheters may play an important role in optimizing VT ablation outcomes in this complex population.
